# Editorial: Neuro-cognition in human movement: from fundamental experiments to bio-inspired innovation

**DOI:** 10.3389/fneur.2026.1625712

**Published:** 2026-01-28

**Authors:** Ramona Ritzmann, Kevin De Pauw, Bettina Wollesen

**Affiliations:** 1 Department of Sport and Sport Science, University of Freiburg, Freiburg, Germany; 2 Innovation Translation Center, AO Foundation, Davos, Switzerland; 3 Department of Physiotherapy, Human Physiology and Anatomy, Faculty of Physical Education and Physiotherapy, Human Physiology and Sports Physiotherapy Research Group (MFYS), Brussels, Belgium; 4 Brussels Human Robotics Research Center (Brubotics), Brussels, Belgium; 5 Institute of Movement Therapy and Movement-oriented Prevention and Rehabilitation, German Sports University Cologne, Cologne, Germany

**Keywords:** sensory-motor integration, motion, motor learning, neuroplasticity, brain-machine interfaces (BMIs), motor control, robotics, cognitive neuroscience

## 1 Introduction

This Editorial provides an in-depth overview of motor execution involving neurocognitive aspects integrating somatosensory inputs and their significance in human movement bridging fundamental research to bio-inspired innovations. Technology transfer of basic scientific evidence into mainstream practice of innovation-inspired application and technology-supported therapy has been catalyzed in the past decade by broad horizontally and vertically structured methodological progression (1). Recent developments of new neurophysiological and imaging techniques or their synthesis currently allow progress in the experimental evaluation and tracking of cognitive or motor short-term and long-term learning which is implemented in transfer arms into practice (1, 2). The advance in technology innovation of the past decade allows not only a broad practical connectivity in technical innovations and digital solutions, but even the purposeful integration of technologies, intelligent solutions, and algorithm-based modeling into active research (3, 4). Examples can be found in smart sensors, feedback-based exoskeletons (5, 6), virtual reality (7), and e-health diagnostics. Still challenging is the validation of the new scientific pathways using experimentally obtained neurocognitive data sets and the global consideration and the dedicated inclusion of these new branches of science in research practice (8).

## 2 Cognition and motor coordination in complex environments

Cognitive control, also known as executive function, refers to the mental processes that allow individuals to regulate their thoughts, emotions, and actions in pursuit of goal-directed behavior (9). In real-world dynamic environments, cognitive control becomes crucial for adapting to changing conditions and achieving desired outcomes despite constant environmental fluctuations (10). These environments—ranging from sports fields to bustling urban settings—demand flexibility, attentional focus, and the ability to switch between tasks or strategies rapidly.

### 2.1 Cognitive control in real-world dynamic environments

In dynamic environments, cognitive control mechanisms are engaged to manage competing demands, resolve conflicts, and plan adaptive responses (11). The prefrontal cortex is particularly central in these processes, serving as the hub for executive functions like inhibition, working memory, and task-switching (12, 13). Furthermore, cognitive control allows for the maintenance of goal-directed behavior despite distractions or unexpected changes in the environment. In the context of sports or robotics, these mechanisms must not only handle internal cognitive load but also coordinate with perceptual processes to align action with external feedback in real-time.

### 2.2 Decision-making and action selection in everyday tasks, and robotics

Decision-making is the process through which individuals choose a course of action from a set of alternatives (14). In robotics, and everyday tasks, decision-making is dynamic and often occurs under conditions of uncertainty and time pressure. Robotics systems, especially in autonomous robots or vehicles, must process a wide range of sensory data to make decisions regarding movement, task execution, and error correction in real-time (15).

The underlying cognitive and neural mechanisms of decision-making in these contexts often involve a balance between automatic, intuitive responses and deliberate, effortful reasoning. Cognitive control plays a role in managing this balance, especially when conditions become uncertain or high-stakes. In everyday tasks, such as crossing a street or driving a car, decision-making integrates sensory inputs with prior experiences, expected outcomes, and risk assessments (16). The efficiency of decision-making can be influenced by factors such as working memory, attention, and the ability to inhibit prepotent or habitual responses (9). The neurobiological mechanisms of decision-making are often studied in the context of reinforcement learning, where the brain learns to optimize actions based on feedback from the environment. Therefore, the sensory input niéeds to be coupled with the motor outputs also in Human Machine Interaction (HMI) (17).

### 2.3 Role of perception-action coupling in movement efficiency

Perception-action coupling refers to the dynamic, reciprocal relationship between sensory inputs and motor outputs that underlie coordinated action (18). The efficiency of movement depends not only on physical factors like strength and agility but also on cognitive factors, such as attention, anticipation, and reaction time. This concept is central to understanding how individuals and robotic systems achieve efficient and adaptive movement in real-time. In real-world tasks, efficient movement requires the integration of perception (e.g., visual, auditory, or tactile input) with motor planning and execution. The ability to couple perception and action allows for anticipatory control, where individuals or robots can predict future states and modify their behavior accordingly.

In robotics, perception-action coupling is crucial for tasks such as navigation, manipulation, and interaction with the environment (19). Autonomous robots use sensors (e.g., cameras, lidar, and force sensors) to gather data about the environment, which is then used to inform their motor actions (20). The coupling of perception and action in robotics systems has advanced significantly with the development of techniques such as computer vision and machine learning, allowing robots to adapt to dynamic environments and optimize movement efficiency in real-time.

The interaction between cognitive control, decision-making, and perception-action coupling is essential for achieving optimal performance in dynamic environments (19, 20). For example, in daily environments, cognitive control mechanisms regulate the decision-making process by managing attention, inhibiting inappropriate responses, and adapting to environmental cues. The coupling of perception and action ensures that the the individual can adjust movements in real-time based on sensory feedback, while decision-making processes guide the selection of appropriate actions in response to changing conditions.

Similarly, in robotics, efficient action selection is facilitated by decision-making algorithms that process sensory input in conjunction with movement commands (3). Cognitive control in this context helps manage resource allocation and action prioritization, while perception-action coupling enables the robot to adjust its movements based on feedback from its sensors. This integration is vital for maintaining efficiency, reducing errors, and improving task performance in environments that are both unpredictable and dynamic. In industrial settings, collaborative robots (cobots) also depend on this coupling. As a worker and robot share the same workspace, their actions must be synchronized in real time. For instance, if the worker is placing a component on an assembly line, the robot must sense the worker's position and actions, adjusting its own movements to ensure safety, efficiency, and the correct handling of tasks. Cognitive control plays a crucial role in these interactions, as the human operator must be aware of both their own movements and the robot's actions, while also interpreting the robot's feedback (21).

## 3 Bio-inspired innovations in technology and robotics

Big potential exist to leverage socioeconomic effects of technological innovation on aging, pathological conditions and shifts in social care.

### 3.1 Socioeconimic impact of new technologies

Assistive technologies, such as AI-powered prosthetics, wearable neurostimulators and exoskeletons, offer new possibilities or individuals with neurodegenerative disorders, reducing healthcare burdens and enabling greater independence (1, 22). In aging societies, smart rehabilitation tools could alleviate pressure on caregivers and improve quality of life (8). However, challenges remain, including accessibility, ethical considerations, and disparities in technology adoption across different socio-economic groups.

Bio-inspired technologies have a major impact on healthcare, assistive robotics, and social care. These innovations hold potential in addressing some of the most pressing socioeconomic challenges arising from demographic aging, chronic health conditions, and the evolving role of caregivers in society (8, 23). It is known that the global population age, which puts pressure on healthcare systems and social care infrastructures. Besides developing adequate fall prevention programs through identifying biological, behavioral, environmental, and socio-economic fall risk factors (24), the rise of bio-inspired robotic systems, such as exoskeletons, soft robotics, and sensor-embedded wearables, are aiding older adults to maintain autonomy, mobility, and social participation (5, 25). By integrating musculoskeletal structures and neuromuscular control, these systems offer more naturalistic support than traditional assistive technologies, thereby reducing dependency (26).

### 3.2 Translating neuro-cognitive principles into bio-inspired robotic systems

Translating neuro-cognitive principles into bio-inspired robotic systems draws from the functioning of the human brain and nervous system to design robots that replicate similar processes (27). For example, neuromorphic systems emulate the brain's neural structures, enabling robots to process sensory data and make decisions like the human brain (27, 28).

Cognitive models such as ACT-R and SOAR simulate human cognition, including reasoning, memory, and problem-solving. By integrating these architectures, robots can perform complex tasks that require reasoning, planning, decision-making, and social interaction (29). Robots can use artificial neural networks (ANNs) and deep learning algorithms for tasks like object recognition, speech processing, and environmental mapping, enabling autonomous navigation in dynamic environments (30). As the brain allocates cognitive resources based on task demands, robots can adjust their cognitive workload depending on task complexity. By prioritizing tasks and adjusting focus, robots can manage multi-step processes more effectively, much like humans shift attention according to environmental demands. This gives potentials to support interactions in rehabilitation or elderly care (31). Moreover, by adding neuro-cognitive motor learning principles involving refining of motor skills through feedback and adaptation, robots can employ reinforcement learning algorith§ms to improve their actions over time, adapting motor commands based on environmental feedback, which is crucial for dynamic tasks like walking and object manipulation (4). This might also be combined sensory feedback (e.g., cameras, accelerometers) to adjust movements in real-time, such as modifying grip strength or avoiding obstacles (32).

Translating neuro-cognitive principles into bio-inspired robotic systems allows robots to not only interact with their environment physically but also process information, learn, and adapt. By mimicking biological processes like learning, perception, and decision-making, robots can function more intuitively, enhancing their utility in fields such as healthcare, manufacturing, and human-robot collaboration.

Current research also showed that intent-driven, intuitive interfaces minimize cognitive load, respond predictively to user intent, and adapt to individual variability in signal patterns (33). Reinforcement learning, and user-in-the-loop design paradigms are enabling prosthetic and robotic devices to adjust in real time to environmental challenges, fatigue, or changes in neural signal quality (26). However, it should be noted that challenges remain. Long-term reliability of neural signal acquisition, especially in non-invasive systems, and inter-individual variability must be addressed. Furthermore, accessibility is important in terms of affordability and scalability. Future perspectives focusing on the convergence of neuro-cognitive science, biomechatronics, AI, and material innovation (e.g., soft robotics, smart textiles) will push neuroprosthetics and BCIs from niche rehabilitation tools toward augmentative technologies, enhancing both impaired and able-bodied performance.

### 3.3 Applications of bio-inspired design in wearable technology and rehabilitation

In clinical rehabilitation, wearable devices such as assistive exosuits, sensor-embedded garments, and smart orthoses are used to support recovery after stroke, spinal cord injury, or orthopedic trauma (34). Their bio-inspired design allows for task-specific training and neuroplasticity-driven motor re-learning (34). These systems enable high-intensity, repetitive practice without requiring constant therapist supervision. By decentralizing care from the clinic to the home, wearable technologies offer new pathways for long-term, self-managed recovery. Additionally, cognitive-motor interaction is increasingly recognized as vital in rehabilitation. Wearables equipped with neurocognitive monitoring tools, such as EEG, can assess and modulate attentional load, dual-task performance, and mental fatigue (35–38), allowing for personalized interventions.

In occupational settings, exosuits have been shown to reduce musculoskeletal strain during lifting or overhead tasks, reducing injury risk while maintaining productivity (6). Bio-inspired wearables can also benefit physical activity and sports performance. In sports science, wearable systems monitor biomechanics and neuromuscular dynamics to optimize performance and prevent overuse injuries (39).

Despite significant advances, it should be mentioned that issues remain such as weight, cost, comfort, and user acceptance, limiting widespread adoption. Moreover, the integration of wearable systems with neurocognitive data raises concerns about privacy, autonomy, and long-term behavioral impacts. Therefore, future research is needed in terms of modular and scalable architectures for individualized use, gender- and age-inclusive design, and multidisciplinary validation frameworks. Furthermore, future developments are moving toward wearables that can adapt to the user and co-evolve with them over time, using reinforcement learning and cloud-based neural networks (40). These will enable personalized baseline tracking, predictive analytics, and context-aware support across diverse environments.

## 4 New insights from the article collection

Within this special issue all the articles focus on how cognitive and motor functions are interconnected, particularly in the context of rehabilitation, external devices, or social interactions. Some studies focus on external devices (i.e., exoskeletons, virtual reality or exergames), while others focus on social dynamics (i.e., partner absence) and how this influence cognitive functioning.

Within wearable technology use, Wollesen et al. aim to evaluate how upper-extremity exoskeletons impact cognitive resources, posture, and muscle synergies during physically and cognitively demanding tasks. This study protocol investigates the interplay between physical assistance and cognitive function, crucial for understanding how technology can optimize human performance while minimizing strain. The researchers plan to use motion analysis (3D), functional near-infrared spectroscopy (fNIRS), and surface electromyography (sEMG) to capture motor performance, muscle activation, and brain activation. By exploring gender-specific effects, the study addresses whether current exoskeleton designs accommodate diverse anthropometric needs, thereby influencing muscle activation and cognitive resource allocation.

Gräf et al. examined how a passive exoskeleton affects both physical and cognitive functions during overhead tasks. They found that using the exoskeleton reduced shoulder muscle activity and improved cognitive performance, especially in dual-task situations following fatigue. Outcomes highlight the relationship between physical support via exoskeletons and neurocognitive resources in demanding tasks.

Büttiker et al. designed a pilot study to explore cognitive-motor exergame training for stroke patients. The study aims to improve cognitive and physical functions through exercises that combine motor tasks and cognitive challenges. The research emphasizes neuroplasticity and the potential of combining cognitive and physical rehabilitation for stroke recovery, targeting the brain-body connection.

Maricot et al. studied the reliability of the Reactive Balance Test (RBT) in individuals with chronic ankle instability (CAI). The tRBT evaluates the role of cognitive processes, like reaction time and accuracy, in maintaining postural stability. Their findings demonstrate that balance performance is closely linked to cognitive processing, making it a useful tool for assessing motor control in rehabilitation in patients with CAI.

Jia et al. explored how the absence of an intimate partner influences cognitive and emotional responses during competitive situations. They found that the lack of a partner enhances interbrain synchronization, particularly in terms of focus and empathy. This study sheds light on how social dynamics affect cognitive processes, such as emotional regulation and cognitive control, during social interactions.

Cui et al. published a study protocol of a single center, evaluator blinded, prospective, two arm parallel group randomized controlled trial with 1:1 allocation ratio in stroke patients. The experiment group receives a combined treatment of 360° VR video action observation and neuromuscular electrical stimulation. The control group receives a cbined treatment of virtual reality landscape observation combined with neuromuscular electrical stimulation. The Fugl-Meyer Assessment for Upper Extremity is the primary outcome of this study, Brunstrom Recovery Stages for Upper Extremity, Manual Muscle Test, Range of Motion, Modified Barthel Index, and Functional Independence Measure are the secondary outcomes. In addition, functional near-infrared spectroscopy (fNIRS) and surface electromyography (sEMG) are used to evaluate the activation of MNS brain regions and related muscles, respectively.

Cognitive and Physical Integration is one topic that aligns with all manuscripts in this special issue. All studies examine how cognitive processes (i.e., such as attention, learning, memory, and decision-making) are influenced by physical tasks or external devices (i.e., exoskeletons, exergames, and perturbation of posture control). Whether it's stroke recovery, balance control, or cognitive enhancement, each study involves some form of rehabilitation or performance improvement that requires both cognitive and motor functioning.

Through measurements like fNIRS, tests for balance, and EEG hyper scanning, these studies highlight the importance of measuring and understanding the relationship between cognitive performance and motor control.

## 5 Challenges and future directions

Current research is still limited due to the lack of translation of fundamental science into scalable, human-centered applications. This gap reflects the need for interdisciplinary methodologies that bridge cognitive science, human movement science, biomedical, and mechanical engineering.

### 5.1 Gaps in current research and the need for interdisciplinary approaches

The core limitations in current research is the unidisciplinary nature of science. While neuroscience has made significant strides in understanding brain plasticity, motor learning, and sensory-motor integration, these insights mainly remain disconnected from robotics, materials science, and/or rehabilitation engineering. This approach hampers the design of technologies that align with the complexity of human behavior, leading to solutions that are functionally effective but cognitively or socially misaligned, e.g., exoskeletons reduce muscle activity at the level of support (41), but still fail to account for mental and cognitive workload, user comfort, and user acceptance (42). These are factors critical for mass adoption of the technology (43).

Much of the research is also conducted in laboratory conditions, offering limited insight into how technologies perform in dynamic, real-world settings. Although there is research showing that lab results cannot be simply transferred to in-field situations (44), and an increasing amount of research is conducted in-field there is still a gap of task and user specific implementations (43). Additionally, current research frequently excludes age, gender, and anthropometric diversity in design and testing, despite the importance for adoption and usability. More recently, there is a push toward inclusion of for example female participants in robotics technology (Wollesen et al.), advancing testing and designing technology for a wider range of individuals.

While the physical aspects of movement assistance have advanced, the cognitive aspects remain underexplored. Technologies that support movement must also support decision-making, attention, emotion regulation, and social interaction. In human-in-the-loop optimization research, there is a push toward the inclusion of user preferences (45). Thus, bio-inspired systems must evolve to reflect the biomechanics of movement and neurocognition, incorporating feedback on mental fatigue, motivation, and executive functioning into the adaptive control strategies of devices and interfaces. To achieve this, research must integrate interdisciplinary co-creation, including interdisciplinary research teams combining neuroscience, robotics, AI, human movement science, ethics, but also living labs and real-world trials to evaluate devices in real-life environments. Furthermore, the integration of standardized protocols and open datasets to facilitate cross-study comparison and reproducibility is needed.

### 5.2 Potential impact of neuro-cognitive innovations on health and industry

In healthcare, neuro-cognitive technologies offer the opportunity for personalized and adaptive interventions to enable individuals with paralysis, stroke, or neurodegenerative diseases to regain control over movement, communication, and daily activities, enhancing autonomy and reducing caregiver dependency. At the same time, wearable systems that integrate neurofeedback, gait analysis, and physiological monitoring allow for continuous, at-home rehabilitation, which is crucial for long-term recovery.

Importantly, these systems allows for neuroplasticity to take place, reinforcing neural reorganization through task-specific training, which accelerates functional restoration and enhances cognitive-motor integration (46). The incorporation of cognitive load monitoring and mental fatigue detection, through for example the use of EEG, into wearable neurotechnologies further supports the development of holistic rehabilitation paradigms. These tools have the potential to reduce healthcare burdens. By decentralizing rehabilitation and enabling early detection of functional decline, neuro-cognitive technologies can reduce the pressure on healthcare services and promote sustainable, community-based care models.

### 5.3 Future trends in neuro-cognitive research and bio-inspired technology development

One of the trends in neurocognitive research and bio-inspired technology is the development of neuro-adaptive systems. These are devices that continuously adjust to the user's cognitive and motor state through real-time interpretation of multimodal neural, physiological, and biomechanical signals (26). Brain-computer interfaces (BCIs) and wearable robotics will increasingly integrate data from EEG, EMG, eye tracking, and IMU's to decode human movement intention, and modulate assistance based on fatigue, focus, stress, and learning progression, creating closed-loop interactions that reflect the dynamic nature of human sensorimotor control. The integration of bidirectional neural interfaces will enable more embodied and intuitive device use, which will advance applications from rehabilitation to augmented cognition.

The next generation of bio-inspired neuro-technologies will be highly personalized. Digital twins will leverage AI and *in-silico* musculoskeletal modeling to simulate rehabilitation outcomes, optimize interventions, and guide training protocols tailored to each individual end-user. These models will allow clinicians and users to predict the effects of therapy, robotics configuration and customized control, or adapt workload on both motor performance and brain adaptation. Deep and reinforcement learning algorithms embedded within robotics devices will allow technologies to allow for customizing robotics control, continuously co-adapting depending on user's preferences and environment. This personalization will benefit user acceptance and long-term engagement. Future research will advance movement assistance in combination with cognitive functioning, such as attention, decision-making, emotional regulation, and social interaction. Devices will be expected to sense and adapt to these higher-order processes, fostering cognitive-physical integration critical for tasks in complex, real-world environments. This shift will probably allow advanced technologies to be used as both assistive and augmentative technologies.

An aspect to be considered in future research is the importance of ethical and legal frameworks. These frameworks should include neural data governance (ownership, consent, and data storage) and risk assessment for cognitive influence and behavioral modification.

## 6 Conclusion: call to action for continued interdisciplinary collaboration and innovation

Future research should foster interdisciplinary collaboration, bridging cognitive neuroscience, human movement science, engineering, and clinical practice. Next steps should also prioritize real-world validation, ensuring innovations move from lab to life, with inclusive design that accounts for gender, age, and functional diversity. Additionally, ethical frameworks should be developed, addressing data privacy, neural autonomy, and long-term impacts of neurotechnologies. Furthermore, research should create open, shared datasets and protocols to support reproducibility, scalability, and faster cross-sector innovation.

This Editorial gives future directions in which science will shape smart, adaptive technologies. By conducting interdisciplinary research, we are capable of developing technologies that can rehabilitate and assist, and even empower human potential in increasingly complex environments.
